# Supplementary material for: Cultivation of Spore-Forming Gut Microbes Using a Combination of Bile Acids and Amino Acids
Source: Microorganisms. 2021 Aug 2;9(8):1651. doi: 10.3390/microorganisms9081651 (PMC8401671; doi:10.3390/microorganisms9081651)
Supplement: Supplementary file 1 [file microorganisms-09-01651-s001.zip › microorganisms-1300112-SI.pdf]

## Supplementary Materials

(Donor A:35 y, male)

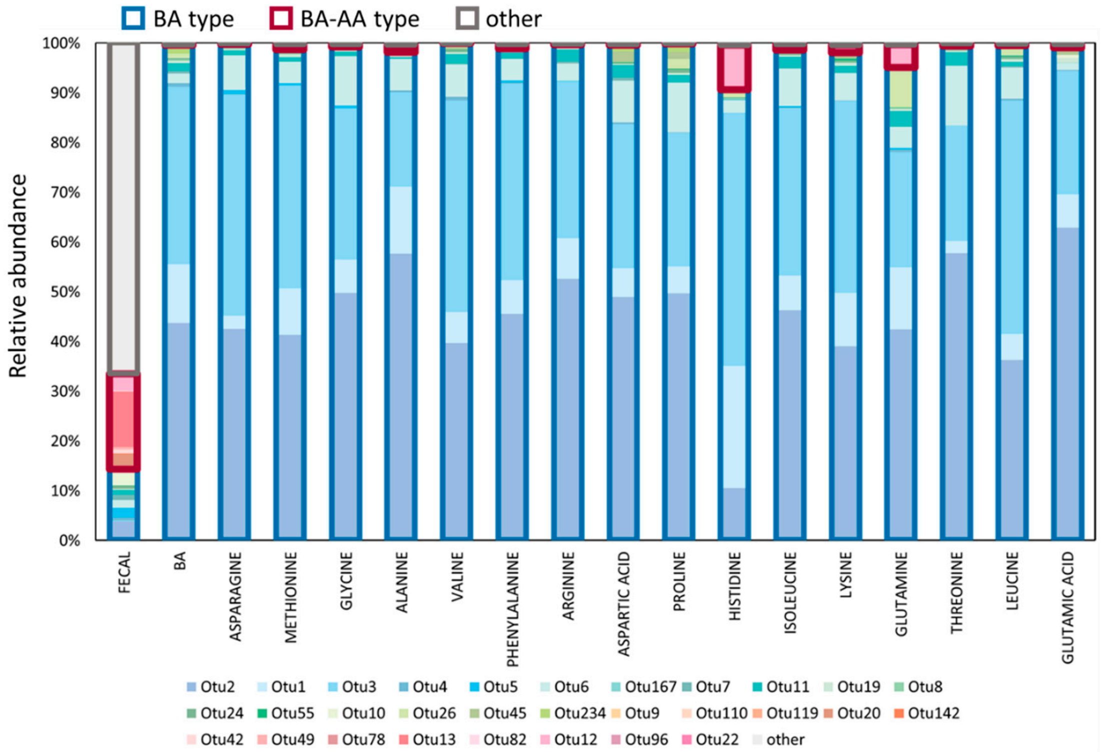


(Donor B:23 y, female)

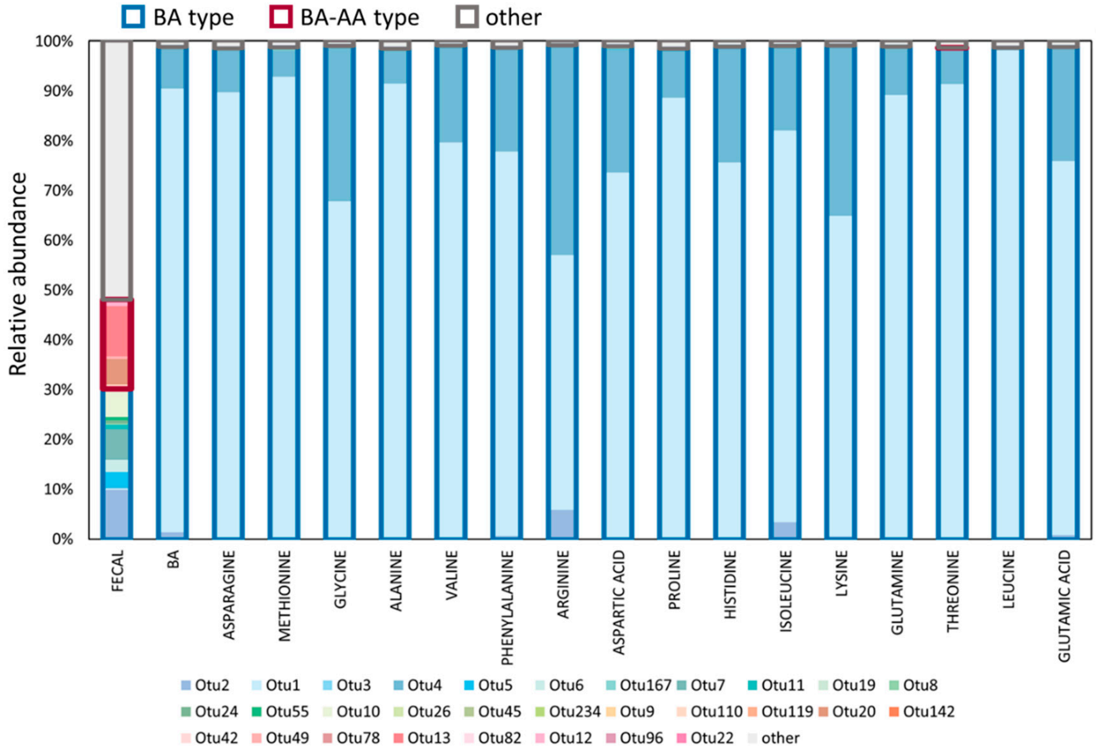

(Donor C:25 y, female)

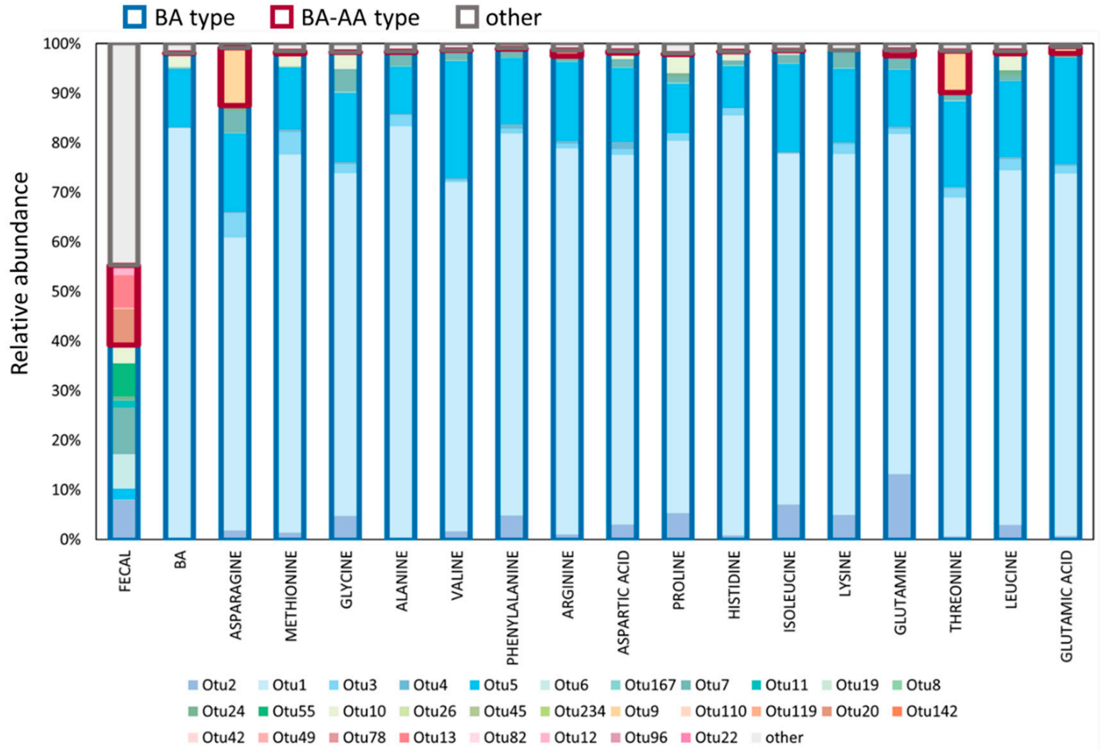


**Figure S1.** The population of the OTUs in each plate. Each panel shows the relative abundance of each OTU observed in each fecal sample (left end) and bacteria colonies recovered from each donor fecal samples treated by each amino acid. Colonies on the plate were pooled and subjected to the 16S rRNA gene amplicon sequencing. Also, to capture the microbiome of original fecal samples, total bacterial DNA was subjected to the 16S rRNA gene amplicon sequencing. OTUs belonging to the BA type are shown in blue, and those belonging to the BA-AA type are shown in red.

**Table S1.** Structural information on bile acids used in this study.

| Code | Name | Structure |
|---|---|---|
| GCA | glycocholic acid | N-(3α,7α,12α-trihydroxy-5β-cholan-24-oyl)glycine |
| GCDCA | glycochenodeoxycholic acid | N-(3α,7α-dihydroxy-5β-cholan-24-oyl)glycine |
| GDCA | glycodeoxycholic acid | N-(3α,12α-dihydroxy-5β-cholan-24-oyl)glycine |
| TCA | taurocholic acid | 2-[(3α,7α,12α-trihydroxy-24-oxo-5β-cholan-24-yl)amino]ethanesulfonic acid |
| TCDCA | taurochenodeoxycholic acid | 2-[(3α,7α-dihydroxy-24-oxo-5β-cholan-24-yl)amino]ethanesulfonic acid |
